# Emotional Distress and Academic Presenteeism in Male University Perpetrators of Intimate Partner Violence: A Mediated Structural Model

**DOI:** 10.3390/bs16060947

**Published:** 2026-06-09

**Authors:** Dennis López-Odar, Arístides Vara-Horna, Zaida Asencios-Gonzalez, Eloína Callejas

**Affiliations:** 1 Facultad de Psicología, Universidad Nacional Federico Villarreal, Lima 15001, Peru; 2 Facultad de Ciencias Administrativas y Recursos Humanos, Universidad de San Martín de Porres, Lima 15011, Peru; avarah@usmp.pe (A.V.-H.); zasenciosg@usmp.pe (Z.A.-G.); 3 Carrera de Administración de Empresas, Universidad Mayor de San Andrés, La Paz 9999, Bolivia; ecallejas@umsa.bo

**Keywords:** violence against women, university students, perpetrators, emotional distress, academic presenteeism, academic performance, mediation model

## Abstract

Although the consequences of intimate partner violence (IPV) for female victims have been widely documented, the psychological and academic correlates of perpetration remain underexplored. This study examines whether emotional distress statistically mediates the association between IPV perpetration and academic presenteeism among male university students. A cross-sectional survey was administered to 343 students from the Universidad Mayor de San Andrés in Bolivia. Using validated instruments and Partial Least Squares Structural Equation Modeling, we assessed direct and indirect associations. Findings indicate that 50.1% of students reported perpetrating at least one form of IPV since entering university, with stalking and psychological violence being most common. Perpetrators reported higher levels of emotional distress compared to non-perpetrators and exhibited higher academic presenteeism (reduced academic functioning despite physical attendance). The structural model indicated a significant indirect statistical effect of IPV perpetration on academic presenteeism through emotional distress (β = 0.137, *p* < 0.001), accounting for 36.2% of the total effect. These findings suggest that universities may consider perpetrator-focused components within broader prevention and support systems, integrating behavioral accountability with screening, referral, and academic support while recognizing that intervention effectiveness was not tested in this study.

## 1. Introduction

Intimate partner violence against women (IPVAW) is a global public health issue with profound implications for individual well-being and social development. Its consequences have been widely documented in various domains, including health, education, and labor productivity (Duvvury et al., 2013; World Health Organization, 2013). Manifesting in psychological, physical, sexual, and economic forms, IPVAW often escalates in cycles of coercion and control, particularly affecting women in heterosexual relationships (Vara-Horna, 2013; Fulu et al., 2013).

In South America, countries such as Peru, Bolivia, and Ecuador report some of the highest IPVAW prevalence rates globally, with lifetime prevalence exceeding 40% (World Health Organization, 2013). Bolivia, in particular, faces alarming rates of violence, with 9 out of 10 women reporting victimization and frequent cases resulting in femicide (Rosales, 2021). While most research has focused on the impact of IPVAW on female survivors, emerging studies suggest that the perpetrators, especially young men, may also experience personal and professional consequences related to their violent behavior (Scott et al., 2017; Stark et al., 2020).

The university context is a particularly important setting for studying male-perpetrated IPV because it concentrates young adults during a developmental period in which intimate relationships, gender norms, peer influence, emotional regulation, and academic trajectories are still being consolidated. Surveys in Peru, Ecuador, Chile, and Mexico indicate high self-reported rates of IPV perpetration among male students, primarily in the form of psychological and stalking behaviors (Vara-Horna & López-Odar, 2016, Vara-Horna, 2021). Despite these findings, very few studies have examined how such behavior is associated with perpetrators’ own academic functioning. Focusing on male university students therefore provides insight into an underexamined group that is relevant for campus prevention, early identification, and student-support policy.

Evidence from the labor sector provides a useful bridge to the academic setting. A growing body of research suggests that men who perpetrate intimate partner violence (IPV) may report emotional disturbances and reduced productivity at work, a phenomenon referred to as presenteeism (Duvvury et al., 2022; Lim et al., 2004). Academic presenteeism, its educational analogue, is defined as a state in which students are physically present but cognitively and emotionally disengaged, leading to diminished performance (Chafloque et al., 2018). Studies in South Africa and Latin American contexts indicate that perpetrators may face concentration difficulties and psychological strain, alongside disrupted academic trajectories (Moruri & Obioha, 2020; Vara-Horna, 2021). However, the psychological mechanisms linking IPV perpetration and academic functioning remain insufficiently explored.

This study aims to fill this gap by investigating the mediating role of emotional distress in the relationship between intimate partner violence (IPV) perpetration and academic presenteeism among university students. Cognitive dissonance theory provides the primary theoretical basis for the tested model, while moral injury and perpetrator-focused trauma perspectives are used as auxiliary interpretive lenses. By testing this statistical mediation model, we aim to extend the literature beyond victim outcomes to examine how perpetration may be linked to students’ psychological strain and academic functioning.

### 1.1. Theoretical Framework

Although emotional distress has traditionally been examined among survivors of intimate partner violence (IPV), a smaller body of work suggests that perpetration may also be associated with psychological strain. The primary theoretical basis of this study is Cognitive Dissonance Theory (Festinger, 1957), which posits that psychological discomfort arises when behavior conflicts with internal standards or self-concept. Applied cautiously to IPV perpetration, this framework suggests that some perpetrators may experience aversive self-evaluative states when reflecting on harm inflicted, which may be expressed as emotional distress.

Moral distress, moral injury, moral trauma, and perpetrator-focused trauma perspectives are treated here as complementary interpretive lenses rather than as mechanisms directly tested in the model. These frameworks help explain why distress responses may be heterogeneous across perpetrators and why severe distress indicators may appear in some profiles (Litz et al., 2022; Mahlako et al., 2023; Mimault et al., 2024; VanderWeele et al., 2025). They are not used to infer clinical diagnosis or causal sequencing from the present cross-sectional data.

Accordingly, this study adopts emotional distress as a unifying operational construct. Emotional distress is used as a non-diagnostic measure of self-reported psychological strain (e.g., negative affect and dysphoric symptoms) that may co-occur with dissonance-related appraisals, moral emotions, or broader psychological burden. This operational focus is better suited for a non-clinical university sample than diagnostic categories and permits a conservative test of the association between IPV perpetration, emotional distress, and academic presenteeism.

The theoretical claim is therefore deliberately limited: IPV perpetration may be statistically associated with emotional distress, and emotional distress may be statistically associated with poorer academic functioning, operationalized here as academic presenteeism. The model tests these associations and the corresponding indirect path; it does not establish temporal order, clinical etiology, or causal mechanisms.

#### 1.1.1. IPVAW Perpetration and Emotional Distress

While the psychological consequences of IPV for victims are well established, research on its emotional effects on perpetrators remains limited. Available studies suggest that IPV perpetration is associated with elevated psychological distress and negative affect, with some reports documenting anxiety-related symptoms and suicidal ideation among specific perpetrator groups (Stark et al., 2020; Vara-Horna et al., 2017). Evidence regarding perpetration-related self-conscious emotions (e.g., guilt and shame) is discussed in the moral injury and self-evaluative emotion studies; accordingly, we treat these emotions as theoretically plausible correlates of distress rather than as empirically established consequences in the present study.

From a theory-building perspective, dissonance and moral-injury frameworks suggest that distress responses may vary depending on perpetrators’ moral awareness, self-evaluative tendencies, coping resources, and broader psychosocial context. Accordingly, we treat perpetration-related distress as a plausible correlate of violent behavior rather than as a uniform consequence.

Consistent with this view, some studies report positive associations between IPV perpetration and mental-health-related indicators, including anxiety-related symptoms and elevated distress in specific perpetrator groups (Mimault et al., 2024; Stark et al., 2020). Therefore, we hypothesize the following:

**Hypothesis 1 (H1).**  *Intimate partner violence perpetration is positively associated with emotional distress.*

#### 1.1.2. Emotional Distress and Academic Presenteeism

Emotional distress is commonly linked to reduced attentional control, lower motivation, and diminished perceived capacity to sustain effort, which may be reflected in poorer academic functioning. In university settings, this pattern is often operationalized as academic presenteeism—attending classes while experiencing psychological strain that is associated with reduced productivity and performance (Chafloque et al., 2018).

Although most existing studies focus on GPA or dropout rates as indicators of academic performance, few have explicitly examined presenteeism as a mediating mechanism. Studies from Latin America and Asia have identified a strong inverse relationship between emotional distress and academic success, often mediated by concentration difficulties and fatigue (Jiang et al., 2022; Franco Mej et al., 2011; Castellanos et al., 2017). Accordingly, we hypothesize the following:

**Hypothesis 2 (H2).**  *Higher emotional distress will be positively associated with higher academic presenteeism.*

#### 1.1.3. Mediation of Emotional Distress

While the association between IPV perpetration and academic functioning has been examined in prior work, the processes that may account for this association remain insufficiently specified. Prior evidence in university samples suggests that health and psychological strain are related to academic performance indicators (Brewer et al., 2018), which motivates the present indirect-effect hypothesis.

Building on the theoretical perspectives outlined above, we propose an indirect association in which IPV perpetration is linked to higher emotional distress, and emotional distress is linked to higher academic presenteeism (Review the Supplementary Materials). Consistent with the cross-sectional design, this mediation is interpreted as a statistical indirect path rather than evidence of temporal or causal ordering. Therefore, we hypothesize the following:

**Hypothesis 3 (H3).**  *Emotional distress statistically mediates the association between intimate partner violence against women (IPVAW) perpetration and academic presenteeism.*

## 2. Materials and Methods

### 2.1. Research Design

This study employed a quantitative, cross-sectional, non-experimental design with a multivariate analytical approach. Given the aim to examine the mediating role of emotional distress in the relationship between intimate partner violence (IPV) perpetration and academic presenteeism, we applied Partial Least Squares Structural Equation Modeling (PLS-SEM) to test the proposed relationships.

### 2.2. Participants

Participants were male undergraduate students enrolled in six academic units at the Universidad Mayor de San Andrés (UMSA), La Paz, Bolivia. A stratified sampling strategy ensured representation across Economics and Finance (40.5%), Law and Political Science (11.7%), Social Sciences (2%), Humanities (2.9%), Engineering (31.8%), Architecture and Arts (10.5%), and Agronomy (0.6%). Of the 501 initial responses, 343 complete and eligible surveys were retained for analysis.

Eligibility criteria included: (a) being enrolled as a university student during the time of data collection, (b) having had at least one romantic or intimate relationship during their university years, including dating, boyfriend/girlfriend relationships, cohabitation, or marriage, and (c) providing informed consent. This criterion was used to ensure that participants had been exposed to a relational context in which the IPV perpetration items were applicable.

The mean participant age was 24 years (SD = 5.71), with a range of 18 to 55 years. A total of 28.6% were in a romantic relationship, 7.9% were married or cohabiting, and 11.1% reported having a disability. Academically, 38.8% were full-time students, and 69.4% had repeated at least one course.

### 2.3. Measuring Instruments and Materials

Data was collected using a self-administered online questionnaire, composed of three validated scales.

#### 2.3.1. Intimate Partner Violence Against Women Perpetration

Assessed via a 12-item scale adapted from Vara-Horna (2021), covering four dimensions: Stalking (2 items), Harassment (2 items), Psychological and economic violence (4 items), and Physical and sexual violence (4 items). Each item used a 7-point frequency scale: 1 = Never, 2 = Happened before but not now, 3 = Once or twice, 4 = 3–5 times, 5 = 6–10 times, 6 = 11–20 times, 7 = More than 20 times. Perpetration was calculated for both the past 12 months and over the whole duration of the relationship.

To enhance reliability and reduce response bias, a bidirectional format was used: participants were asked about both the violence they committed and the violence they received, although only the perpetration data were analyzed in this study.

#### 2.3.2. Emotional Distress

Measured using a 7-item scale adapted from Vara-Horna et al. (2017), evaluating emotional symptoms experienced in the past 12 months (e.g., anxiety, hopelessness, anger, suicidal ideation). Items were rated on a 6-point frequency scale, ranging from 1 (Never) to 6 (More than 20 times).

#### 2.3.3. Academic Presenteeism

Defined as diminished academic functioning despite physical attendance. Measured using 11 items from Vara-Horna and López-Odar (2016), grouped into two dimensions: Distraction and exhaustion (9 items), and Consequences of neglect (2 items). Items were rated on a 6-point scale based on frequency over the past 4 weeks, ranging from 1 (Never) to 6 (More than 10 days). 

### 2.4. Procedure

The study was evaluated and approved by an international committee of experts within the framework of the COTRIA project, a triangular cooperation initiative involving academic institutions from Peru and Bolivia, together with the German Cooperation (GIZ Bolivia, La Paz, Bolivia). All methodological and ethical aspects were reviewed and endorsed by this committee. The research was conducted in strict accordance with the ethical principles of the Declaration of Helsinki (World Medical Association, 2024). The questionnaire was distributed via institutional email using SurveyMonkey and Google Forms, with support from the IT units of each academic unit. Participation was voluntary and anonymous. Informed consent was obtained digitally prior to commencing the survey.

### 2.5. Data Analysis

Data from both platforms were consolidated and cleaned in Excel, then analyzed using SPSS version 28 and SmartPLS 4. Descriptive statistics, chi-square tests, and odds ratios were computed in SPSS to compare perpetrator vs. non-perpetrator groups. PLS-SEM was used to estimate the measurement and structural models because the study was prediction-oriented and exploratory, included hierarchical constructs, and involved non-normally distributed self-report indicators. We acknowledge that covariance-based SEM would also be a defensible alternative for future confirmatory replications of this relatively parsimonious mediation model.

The mediation analysis evaluated direct, indirect, and total statistical effects. To interpret the model, we considered standardized beta coefficients, coefficients of determination (R^2^), effect sizes (f^2^), reliability, convergent validity, and discriminant validity. Statistical significance was evaluated using bootstrapping with 5000 subsamples. Because the design was cross-sectional and non-experimental, direct and indirect effects are interpreted as model-based statistical associations rather than evidence of causal ordering.

## 3. Results

### 3.1. Reliability and Validity

Both reliability and validity were assessed using the Partial Least Squares Structural Equations (PLS-SEM) technique. Regarding reliability, according to Hair et al. (2017), coefficients should be greater than or equal to 0.70 to demonstrate internal consistency among the indicators. Therefore, as shown in Table 1, reliability is established because the composite reliability coefficients range from 0.806 to 0.928.

To establish validity, convergent and discriminant validity were evaluated. Indicator loadings were interpreted in relation to the conventional 0.708 benchmark, and average variance extracted (AVE) was expected to be at least 0.50. As shown in Table 1, most loadings exceeded the expected level; lower-loading indicators were retained when their removal did not materially improve AVE and when they represented substantively important facets of the constructs, especially low-frequency but clinically relevant distress indicators. Thus, the measurement model was considered acceptable, but the lower-loading indicators should be interpreted with caution.

Regarding discriminant validity, the Fornell–Larcker criterion and the heterotrait-monotrait ratio (HTMT) were evaluated (Table 2). The Fornell–Larcker criterion indicated that the square root of the AVE shared between each construct and its indicators was greater (Academic Presenteeism = 0.709; Emotional Distress = 0.743; IPVAW = 0.663) than the correlations with other constructs (Academic Presenteeism and Emotional Distress = 0.623; Academic Presenteeism and IPVAW = 0.238; Emotional Distress and IPVAW = 0.223). In addition, HTMT values were below the conservative 0.85 threshold (Hair et al., 2017), supporting discriminant validity among the main constructs.

### 3.2. Analysis of IPVAW Prevalence

Among the 343 participants (Table 3), 50.1% reported having perpetrated at least one form of intimate partner violence (IPV) during their university years. In the past 12 months, 25.9% admitted to having committed IPV, with an average of 6.8 incidents (SD = 11.3). The most frequently reported forms were stalking (29.2%) and psychological violence (28.1%), followed by sexual violence (11.2%), physical violence (10.9%), and economic violence (8.4%). These prevalence rates are high and should be interpreted as self-reported perpetration in a sensitive survey context rather than as institutional prevalence estimates. They nonetheless indicate that psychological and stalking-related behaviors are not marginal in this sample and deserve careful discussion in relation to campus prevention and support systems.

### 3.3. Emotional Distress in Perpetrators vs. Non-Perpetrators

Students who had perpetrated IPV reported significantly higher levels of emotional distress than non-perpetrators. Table 4 presents group differences across individual symptoms. Perpetrators were more likely to feel overwhelmed, anxious, isolated, and hopeless. Notably, 42.4% reported suicidal thoughts, and 19.2% attempted suicide, figures markedly higher than those of non-perpetrators. Odds ratios for emotional distress indicators ranged from 2.08 to 4.08, reflecting a consistent and robust association between IPV perpetration and psychological strain.

### 3.4. Academic Presenteeism Among Perpetrators

IPV perpetrators also reported significantly higher levels of academic presenteeism. As shown in Table 5, they experienced more frequent distraction during classes, emotional exhaustion, and reduced academic productivity. They were also more likely to make mistakes, fall behind, be reprimanded by professors, or face conflict in group work due to poor engagement. The strongest associations were observed for: Mistakes due to distress (OR = 3.52), Reprimands from instructors (OR = 3.36), and Avoidance of academic obligations (OR = 3.22).

### 3.5. Correlation Between Key Variables

Table 6 presents Pearson correlation coefficients between the main variables. IPVAW perpetration was significantly correlated with emotional distress (r = 0.296, *p* < 0.001) and academic presenteeism (r = 0.334, *p* < 0.001). Emotional distress exhibited a strong positive correlation with presenteeism (r = 0.584, *p* < 0.001), supporting its hypothesized mediating role. 

### 3.6. Structural Model Results

The structural equation model indicated that IPV perpetration was positively associated with emotional distress (β = 0.228, *p* < 0.001), although the effect size was small (f^2^ = 0.055). This relationship explained 5.2% of the variance in emotional distress. In turn, emotional distress was positively associated with academic presenteeism (β = 0.600, *p* < 0.001), with a large effect size (f^2^ = 0.567), explaining 36% of the variance. These results support H1 and H2 as statistical associations (Table 7).

The mediation analysis indicated a significant indirect statistical association between IPVAW perpetration and academic presenteeism through emotional distress (β = 0.137, *p* < 0.001). In the estimated model, emotional distress accounted for 36.2% of the total statistical effect, while the remaining 63.8% corresponded to the direct path. These estimates should be interpreted as cross-sectional model parameters, not as evidence that perpetration temporally causes distress or that distress temporally causes presenteeism.

This supports H3, indicating that emotional distress represents a statistically meaningful indirect pathway linking IPV perpetration and academic presenteeism in the model. Longitudinal or experimental designs would be required to test temporal or causal mechanisms.

## 4. Discussion

This study contributes evidence on an underexamined issue: the psychological and academic correlates of IPV perpetration among male university students. Rather than framing perpetrators as unaffected agents or as passive victims of their own behavior, the findings show that perpetration co-occurred with elevated emotional distress and poorer self-reported academic functioning in this sample.

The prevalence pattern is consistent with prior university-based research in Latin America showing that psychological aggression and stalking-related behaviors are among the most frequently reported forms of male-perpetrated IPV (Vara-Horna & López-Odar, 2016, Vara-Horna, 2021). The present study extends that literature by linking these behaviors to indicators of emotional distress and academic presenteeism, while retaining a non-causal interpretation.

These findings also partially parallel victim-focused university research, where IPV exposure has been associated with anxiety, depressive symptoms, concentration problems, and poorer academic performance (Barroso-Corroto et al., 2022; Brewer et al., 2018). The comparison should be made cautiously because victimization and perpetration are ethically and analytically distinct positions in the violent relationship. Nevertheless, both bodies of evidence suggest that IPV is linked to psychological strain and functional impairment within educational settings. Evidence on female perpetrators is more limited and context-dependent; sex- and gender-disaggregated studies indicate that perpetration can also co-occur with mental-health indicators among young people, but the present male-only sample does not allow direct comparison with female perpetrators (Stark et al., 2020).

The association between perpetration and emotional distress is broadly consistent with studies reporting mental-health-related correlates among perpetrators or violent youth populations (Stark et al., 2020; Mimault et al., 2024). Cognitive dissonance provides the primary interpretive frame: when violent behavior conflicts with self-concept or moral standards, some individuals may report aversive self-evaluative states. Moral injury and perpetrator-focused trauma perspectives help interpret possible heterogeneity in distress, but they remain auxiliary lenses rather than mechanisms directly tested here.

The association between emotional distress and academic presenteeism is also consistent with research on psychological strain, reduced concentration, fatigue, and productivity loss in educational and occupational settings (Chafloque et al., 2018; Duvvury et al., 2013; Jiang et al., 2022). In this sample, perpetrators more frequently reported distraction, exhaustion, mistakes, missed assignments, reprimands, and conflict in group work, suggesting that academic presenteeism may be a useful functional indicator in campus prevention and support systems.

The indirect path through emotional distress was statistically significant and accounted for over one-third of the total model effect. This result supports the plausibility of a psychological-strain pathway, but it should be read as a statistical mediation in cross-sectional data. Alternative explanations remain possible, including prior distress, relationship conflict, substance use, family background, social norms, and other unmeasured factors.

A critical finding is the elevated prevalence of severe risk indicators among perpetrators: 42.4% reported suicidal thoughts and 19.2% reported suicide attempts. Because emotional distress was defined as an operational, non-diagnostic construct, these indicators should be interpreted as markers of high subjective burden rather than as evidence of formal psychiatric diagnosis or as consequences attributable to perpetration alone.

Ethically, these findings indicate that research and institutional responses involving perpetrators should include clear referral pathways and risk-management protocols when severe distress indicators are detected. This does not reduce accountability for violent behavior; it recognizes that comprehensive campus responses may need to combine accountability, victim safety, assessment, referral, and academic support.

Taken together, the findings support a cautious conclusion: in this university sample, IPV perpetration was associated with emotional distress and academic presenteeism. The study does not test intervention effectiveness, but it identifies correlates that universities may consider when designing integrated prevention and student-support strategies.

## 5. Conclusions, Implications and Limitations

This study found evidence consistent with emotional distress statistically mediating the association between intimate partner violence perpetration and academic presenteeism among male university students. In this sample, perpetrators reported higher levels of emotional distress and higher academic presenteeism—manifested as reduced self-reported academic engagement despite physical classroom attendance. Overall, the findings are consistent with a model in which perpetration co-occurs with psychological strain that is linked to academic functioning difficulties, highlighting an underexamined correlation of perpetration within university settings.

### 5.1. Practical Implications

These findings have implications for university violence prevention policies and student support services. Many campus responses prioritize victim services and disciplinary action, while offering limited structured pathways for individuals who have perpetrated violence to engage in accountability-centered change. Given the high self-reported prevalence observed in this sample, universities may need a more comprehensive response that addresses prevention, victim safety, perpetrator accountability, psychological risk, and academic functioning together.

For perpetrators, institutions could consider accessible, low-threshold programs focused on accountability, including recognition of harm, responsibility-taking, emotional regulation, and nonviolent relationship skills. These programs should not replace disciplinary procedures or victim protection; rather, they could complement them when integrated with screening and referral systems for elevated emotional distress, especially when violent behavior co-occurs with academic presenteeism.

For affected partners and other persons involved, universities should strengthen confidential reporting channels, trauma-informed counseling, safety planning, referral pathways, and academic accommodations when needed. Interventions should also consider peer and couple-level educational components only when appropriate and safe, avoiding any approach that pressures victims to participate in joint processes or shifts responsibility away from perpetrators.

At the institutional level, academic presenteeism may be treated as one possible early-warning signal when it appears together with behavioral or emotional risk indicators. This does not mean that presenteeism identifies IPV perpetration; rather, it may provide a non-stigmatizing entry point for outreach, assessment, and referral within existing student well-being systems.

Finally, because the present study identifies associations rather than causal effects, these recommendations should be understood as policy-relevant implications rather than tested interventions. Their effectiveness for improving campus safety, student well-being, or academic performance should be evaluated through longitudinal and intervention research.

### 5.2. Limitations and Future Research

This study has several limitations. First, the sample was drawn from a single university in Bolivia, which may limit the generalizability of the findings to other institutional and cultural contexts. Second, the cross-sectional, self-report design does not allow causal inference or temporal ordering among IPV perpetration, emotional distress, and academic presenteeism. Third, because all variables were measured through self-report at a single time point, the findings may be affected by common-method bias, recall bias, social desirability, defensive underreporting, and self-presentation concerns. This is especially important in research on IPV perpetration, where respondents may minimize or fail to disclose violent behavior. The bidirectional questionnaire format may have reduced some response defensiveness, but it cannot eliminate these biases. Fourth, although the data were consistent with the proposed mediation model, alternative explanations remain plausible. Unmeasured variables—such as substance use, family background, prior trauma exposure, peer norms, relationship characteristics, moral disengagement, and perceived stigma—could influence both perpetration and distress, thereby contributing to the observed associations.

Future research should replicate the model across diverse universities and countries and prioritize longitudinal or intensive repeated-measures designs to evaluate temporal dynamics and strengthen causal inference. Where feasible, studies should incorporate multi-informant or administrative indicators (e.g., academic records, disciplinary reports, counseling utilization, or disciplinary referrals), as well as measures of social desirability and response defensiveness, to reduce common-method bias. Future studies should also compare PLS-SEM and covariance-based SEM specifications and examine moderators that may condition these associations—such as masculinity norms, moral disengagement, emotion regulation skills, and social support—to identify for whom and under what conditions perpetration co-occurs with distress and academic functioning difficulties.

## Figures and Tables

**Table 1:** Validity and reliability of variables.

Items	Loadings	Reliability	AVE
Academic Presenteeism (Second order)		0.738	0.610
Distraction and Exhaustion *(DA)*	0.670–0.859	0.928	0.590
Consequences of negligence (NC)	0.845–0.861	0.842	0.728
IPVAW Perpetration (Second order)		0.867	0.622
Stalking	0.791–0.852	0.861	0.610
Harassment	0.796–0.861		
Psychological and economic	0.713–0.865	0.892	0.553
Physical and sexual	0.657–0.861	0.738	0.610
Emotional Distress	0.426–0.874	0.842	0.728

**Table 2:** HTMT ratios among main constructs.

Construct	IPVAW	Emotional Distress	Academic Presenteeism
IPVAW	1.000	0.178	0.551
Emotional Distress	0.178	1.000	0.743
Academic Presenteeism	0.551	0.743	1.000

Note. HTMT values were computed from the manuscript domain indicators using the reproducible analysis script supplied with the revision package. Values below 0.85 support discriminant validity.

**Table 3:** Prevalence of IPVAW Perpetration.

	During the Relationship (%)	Last Twelve Months (%)
Stalking “To make your partner or former partner fear for his or her personal safety …”	29.2	13.4
You made-in an insistent manner-unwanted phone calls, sent emails, voice or text messages, or posted messages, images, or videos on social networks.	21.4	10.9
You have followed her, watched her, spied on her, waited for her or appeared to her-recurrently.	16.1	5.3
Harassment	20.1	9.8
You have made offensive and demeaning comments about your body, appearance or sexuality, sexually insulting things, or tried to pressure her to talk about sex when she didn’t want to.	15.4	7.7
You emailed, texted, tweeted, called, or sent her offensive sexual messages, jokes, stories, pictures, or sexual videos that she did not want.	11.6	5.1
Psychological violence	28.1	14.6
You have tried to control her by preventing her from going to classes, parties, study meetings, or doing your homework or chores; not allowing her to see or talk to friends or family; or demanding that she dress your way.	22.5	11.1
You have insulted her (…), humiliated her in front of others (…), or insulted or defamed her in public or social networks.	10.6	6.4
You have threatened her with physical distress, or distress to someone else or to yourself, if she does not do what you ask.	5.5	3.0
Economic violence		
You have appropriated her things, taken her money or cell phone, or destroyed her things, books or notebooks, or blackmailed her to destroy them if she does not do what you want.	8.4	4.2
Physical violence		
You have used physical force against her (e.g., bent her fingers, bitten her or pulled her hair; slapped, kicked, or hit her with objects (…); attacked her with a weapon or physically hurt her).	10.9	4.9
Sexual violence.	11.2	5.4
You have kissed, groped, or otherwise sexually touched her against her will and desire (e.g., by using force, threats or blackmail or by taking advantage of her being unconscious or passed out).	8.6	4.0
You attempted to force her to have sex against her will and desire (e.g., by using force, threats, or blackmail, etc.).	6.2	2.5
You forced her to have sex against her will and desire (Using force, threats, or blackmail when she was unconscious).	4.9	2.8
Total prevalence	50.1	25.9

**Table 4:** Emotional Distress in perpetrator and non-perpetrator students (percentages).

Emotional Distress Indicators	Total	Non-Perpetrator	Perpetrator	X^2^	OR	95% CI
Helpless, fearful, or distressed	70.3	57.3	83.1	27.380 ***	3.67	[2.23; 6.06]
Depressed, discouraged, without hope	81.9	76.9	87.2	6.508 *	2.08	[1.18; 3.68]
Stressed, anxious, overwhelmed.	91.8	87.1	96.5	10.057 **	4.09	[1.61; 10.35]
Solitary, isolated, unsupported	77.8	67.8	87.8	19.796 ***	3.41	[1.95; 5.96]
Much anger, rage or intense anger	77.3	70.2	84.3	9.740 **	2.28	[1.35; 3.86]
Suicidal thoughts.	30.9	19.3	42.4	21.509 ***	3.08	[1.90; 5.01]
Suicide attempts	14.3	9.4	19.2	6.766 *	2.30	[1.21; 4.36]
Total	63.5	55.4	71.5			

* *p* < 0.05, ** *p* < 0.01, *** *p* < 0.001.

**Table 5:** Academic Presenteeism in perpetrator and non-perpetrator students (percentages).

Academic Presenteeism Indicators	Total	Non-Perpetrator	Perpetrator	X^2^	OR	95% CI
I was unable to concentrate on studies.	88.0	86.0	90.1	1.404	1.49	[0.77; 2.88]
I was distracted in class.	92.4	91.2	93.6	0.691	1.41	[0.63; 3.15]
Studied slower than usual.	85.4	81.3	89.5	4.685 *	1.97	[1.06; 3.67]
I was tired, exhausted or exhausted while in class or studying.	91.5	87.1	95.9	8.571 **	3.48	[1.45; 8.38]
Had personal or family concerns that affected his studies.	86.9	80.1	93.6	13.686 ***	3.63	[1.77; 7.44]
His performance decreased too much.	86.6	80.1	93.0	12.300 ***	3.31	[1.65; 6.64]
He had no energy to study.	90.1	86.5	93.6	4.779 *	2.28	[1.07; 4.83]
Made mistakes on tests or homework because he was worried or something was bothering him.	83.1	74.9	91.3	16.465 ***	3.52	[1.87; 6.62]
Did not submit work and assignments because was worried or something was bothering him.	71.4	64.3	78.5	8.426 **	2.02	[1.25; 3.27]
Have been scolded or reprimanded by teachers for poor performance.	43.7	29.2	58.1	29.104 ***	3.36	[2.15; 5.26]
I had arguments with classmates for not complying with group work.	42.3	28.1	56.4	28.192 ***	3.31	[2.114; 5.2]
Total	78.3	71.7	84.9			

* *p* < 0.05, ** *p* < 0.01, *** *p* < 0.001.

**Table 6:** Correlation of variables.

Variables	*N*	*M*	*SD*	*g*1	*g*2	1	2	3
IPVAW Perpetration	343	3.8	7.2	3.3	13.7	-		
Emotional distress	343	25.8	26.3	1.3	0.9	0.296 **	-	
Academic presenteeism	343	13.1	9.8	1.1	0.9	0.334 **	0.584 **	-

Note. *N* = number of cases; *M* = arithmetic mean; *SD* = standard deviation; *g*1 = asymmetry; *g*2 = kurtosis. ** *p* < 0.01.

**Table 7:** Direct and indirect statistical effects in the structural model.

Hypotheses	β	t-Values	Bootstrap 95% CI	*p* Values	Decision	R^2^	f^2^
H_1_: IPVAW Perpetration => Emotional Distress	0.228	5.363	[0.164; 0.329]	0.000	Supported	0.052	0.055
H_2_: Emotional distress => Academic Presenteeism	0.600	14.691	[0.521; 0.681]	0.000	Supported	0.360	0.567
H_3_: IPVAW Perpetration => Emotional Distress => Academic Presenteeism	0.137	5.009	[0.097; 0.203]	0.000	Supported		

Note. t-values, *p*-values, and 95% confidence intervals were obtained by Bootstrapping simulation (resampling 5000 subsamples).

## Data Availability

The original data presented in the study are openly available on https://osf.io/db5eg/overview (accessed on 20 February 2026).

## Supplementary Materials

The following supporting information can be downloaded at: https://www.mdpi.com/article/10.3390/bs16060947/s1, Figure S1: Mediation model of emotional distress in the relationship between ipv perpetration and academic presenteeism.
