# Supplementary material for: Emotional Distress and Academic Presenteeism in Male University Perpetrators of Intimate Partner Violence: A Mediated Structural Model
Source: Behav Sci (Basel). 2026 Jun 9;16(6):947. doi: 10.3390/bs16060947 (PMC13295792; doi:10.3390/bs16060947)

Supplementary material

Figure S1. Mediation Model of Emotional Distress in the Relationship between IPV Perpetration and Academic Presenteeism

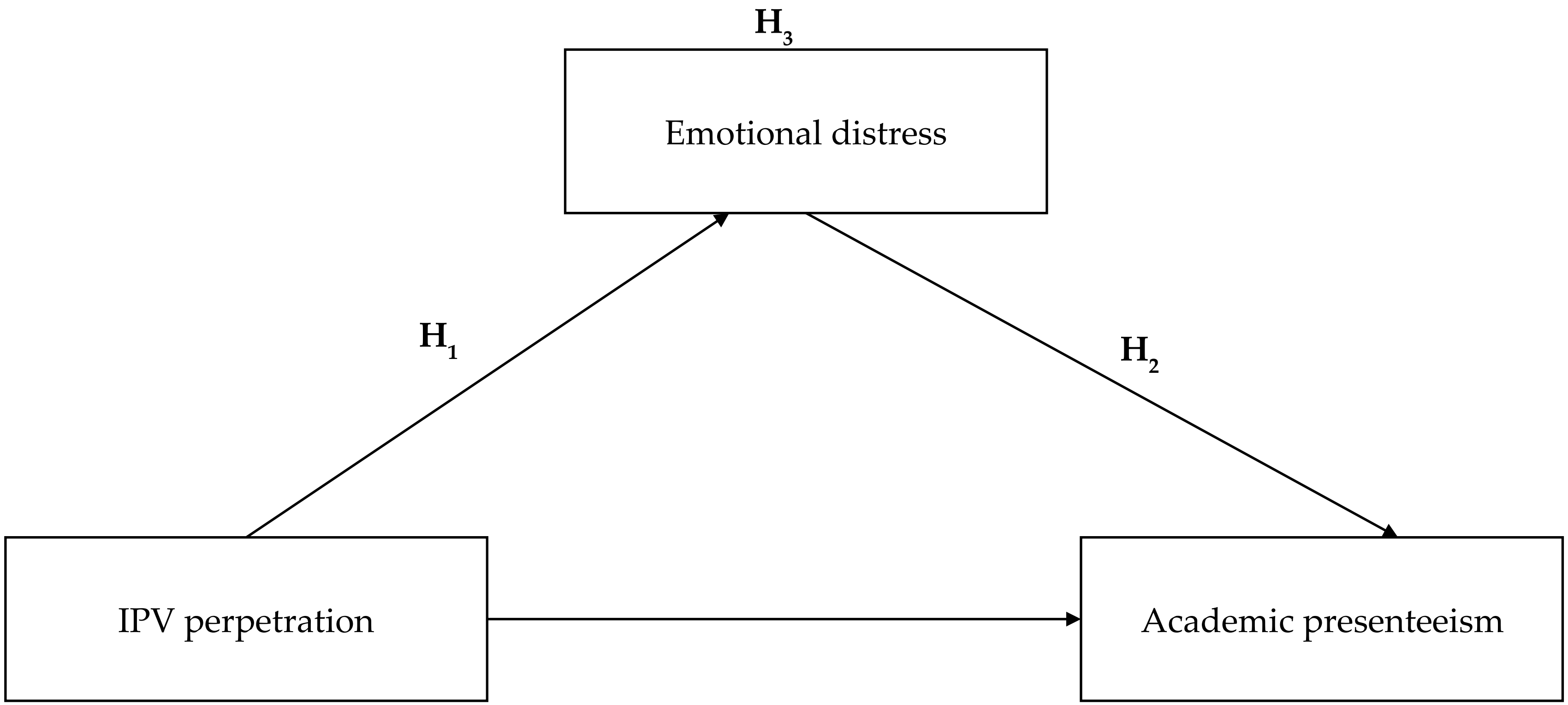

Supplement: Supplementary file 1 [file behavsci-16-00947-s001.zip › behavsci-4191193-supplementary.pdf]
